# A Duplicated, Truncated *amh* Gene Is Involved in Male Sex Determination in an Old World Silverside

**DOI:** 10.1534/g3.117.042697

**Published:** 2017-06-13

**Authors:** Dilip Kumar Bej, Kaho Miyoshi, Ricardo S. Hattori, Carlos A. Strüssmann, Yoji Yamamoto

**Affiliations:** * Graduate School of Marine Science and Technology, Tokyo University of Marine Science and Technology, 108-8477, Japan; † Unidade de Pesquisa e Desenvolvimento de Campos do Jordão, Agência Paulista de Tecnologia dos Agronegócios/Secretaria de Agricultura e Abastecimento, 12460-000, Brazil

**Keywords:** Atheriniformes, *amhy*, fish, *Hypoatherina tsurugae*, sex determination

## Abstract

A master sex-determining gene, the Y chromosome-linked anti-Müllerian hormone (*amhy*) gene, has been described in two New World atheriniform species but little is known on the distribution, evolution, and function(s) of this gene in other Atheriniformes. Interestingly, *amhy* has been found to coexist with temperature-dependent sex determination (TSD), providing a unique opportunity to explore the interplay between genotypic and environmental sex determination. In this study, the search for an *amhy* homolog was extended to an Old World atheriniform, the cobaltcap silverside *Hypoatherina tsurugae* (Atherinidae). The full sequences, including the coding and noncoding regions, of the autosomal *amh* (*amha*) and a putative *amhy* were obtained. The deduced Amha and Amhy proteins comprised 511 and 340 amino acids (aa), respectively. PCR analysis with genomic DNA from wild adults and from laboratory-reared juveniles revealed a high, but not complete association of ∼95% between *amhy* and maleness. The spatiotemporal expression of *amhy* and *amha* during gonadal sex differentiation was analyzed by qRT-PCR and *in situ* hybridization (ISH). *amhy* transcription (in *amhy*-positive larvae) started before and peaked during histological differentiation of the gonads whereas *amha* was negligible during the same period in both genotypes. These results demonstrate that the *amhy*, although with some structural differences in relation to the *amhy* of some New World atheriniforms, is strongly associated with maleness and probably important for testicular development in this Old World atheriniform. Thus, *amhy* is a candidate sex determination gene in cobaltcap silverside and it will be key to scrutinize the mechanism of sex determination in this species.

In recent years, a growing number of genes have been identified as major triggers of sex determination in teleosts (Hattori *et al.* 2013; Kikuchi and Hamaguchi 2013; Takehana *et al.* 2014; and other references below). It is now evident that sex-determining genes in fishes are not restricted to transcription factors as there are reports also implicating members of the TGF-β superfamily and even an immune-related gene with this function. Interestingly, the degree of conservation of these genes apparently varies greatly with the taxonomic group. For instance, while most salmonids share a common sex-determining gene (*e.g.*, *sdY*, Yano *et al.* 2012, 2013), in species of the genus *Oryzias* there are a variety of genes with this function (*e.g.*, *dmy/dmrt1bY*, Matsuda *et al.* 2002; *gsdfY*, Myosho *et al.* 2012; *sox3Y*, Takehana *et al.* 2014).

In silversides, a homolog of the Y chromosome-linked duplication of the *amhy* gene, first discovered in Patagonian pejerrey (*Odontesthes hatcheri*; Atherinopsidae; Hattori *et al.* 2012), was found to be functional also in the congeneric species *O. bonariensis* (Yamamoto *et al.* 2014). Nevertheless, gonadal fate in these two species is affected also by the temperature experienced during a critical period of sex determination early in life (TSD; Strüssmann *et al.* 1997), sometimes overriding the genetic predisposition of an individual and giving rise to phenotypic–genotypic mismatches in relation to *amhy* even at environmentally relevant temperatures. These facts demonstrate the coexistence of two opposing sex determination systems in this group of fishes and suggest that their genetic trigger of sex determination may be functional only within a given thermal range (Yamamoto *et al.* 2014). TSD has also been reported in several other atherinopsids (genera *Menidia* and *Chirostoma*; Strüssmann *et al.* 2010; Corona-Herrera *et al.* 2016) and is conceivably widespread in this taxon (Strüssmann and Patiño 1999). However, in contrast to the abundance of information on atherinopsids, there is little or no knowledge on the sex determination system and evolution of the *amhy* gene in other atheriniform families.

The phylogenetic relationships within Atheriniformes are still controversial and different authors describe it as containing between six and nine families (Sparks and Smith 2004; Nelson 2006; Froese *et al.* 2012). Until recently, atherinopsids (known as “New World silversides”) were included in the family Atherinidae, which is now reserved for the “Old World silversides.” These two families comprise numerous species that inhabit coastal marine, estuarine, and freshwater environments. They are generally small-sized and form large schools that represent an important forage for upper trophic predators worldwide (Bloom *et al.* 2012). This fundamental role in coastal ecosystems and the possibility that many of them display TSD, which renders species particularly vulnerable to global warming and changing climatic patterns (Janzen 1994; Miller *et al.* 2004; Hulin *et al.* 2009), make it urgent that we obtain knowledge of their sex-determining systems and monitor possible environmental effects on population sex ratios and stability.

In this study, we probed the presence of the *amhy* gene and its role in testis determination in a population of the cobaltcap silverside *Hypoatherina tsurugae*, a marine atherinid from the Northwest Pacific Ocean. This study was conceived with two basic aims. The first was to expand our understanding on the distribution of *amhy* among atheriniforms. The second was to provide the scientific basis for using *amhy* as a marker of genetic sex tendency in studies on the molecular mechanism of sex determination of this species.

## Materials and Methods

### Collection of wild specimens

Sexually mature adult cobaltcap silversides were collected by hand net on July 2014 in Tokyo Bay (Chiba, Japan). The gonadal sex of 81 individuals was assessed by careful stripping of gametes and eight fish of each sex were randomly selected for cloning of *amh* genes (see details below). The remaining fish (48 females and 17 males) were stocked in a 500 L circular tank at the Tateyama Station, Field Science Center of Tokyo University of Marine Science and Technology (Chiba, Japan) and used as broodstock fish to obtain gametes and offspring for further experiments (see below).

### Cloning of autosomal amh (amha) and Y chromosome-linked amh (amhy)

Genomic DNA was extracted from the caudal fin tissue of one sexually mature male following the protocol described by Aljanabi and Martinez (1997) and subjected to PCR amplification using degenerate primers designed based on *O. hatcheri amha*. To determine the complete open reading frame for cobaltcap silverside *amha*, total RNA was isolated from testis using TRIzol (Thermo Fisher Scientific, Waltham, MA) following the manufacturer’s instructions and 1 µg of total RNA per sample was reverse transcribed using SuperScript III (Thermo Fisher Scientific) with Oligo-(dT) primers (Merk Millipore, Darmstadt, Germany) in 20 μl reactions. RT-PCR, genome walking, and 5′- and 3′-RACE PCRs using a Smart RACE cDNA amplification kit (Takara Bio, Shiga, Japan) were then performed according to the manufacturer’s protocol. The PCR conditions and specific primers used in each reaction are listed in Supplemental Material, Table S1 and Table S2 in File S1.

Based on the *amha* full sequence, several primer sets flanking intronic sequences were designed in coding regions and used to amplify a Y chromosome-linked *amhy* in this species. This strategy was based on the differences between *amhy* and *amha* genes in *O. hatcheri* and *O. bonariensis*, whereby an insertion of ∼500 bases specific to *amhy* is found in the third intron. Genomic DNA was isolated from the caudal fin of 16 adult fish, eight females and eight males, following the protocol described above and subjected to PCR amplification. One set of the primers designed in the first and fifth exons (Table S1 in File S1; Amh 613 F and Amh 35 R) amplified two fragments. The larger fragment was present in both sexes whereas the smaller one, a putative *amhy*, was present only in males. The smaller fragment was purified, cloned, and sequenced as described above. To obtain the full genomic and cDNA sequences of the putative *amhy*, genome walking and RACE PCR were conducted according to the same protocols as for *amha* cloning.

The specific amplicons from each PCR reaction were purified, cloned, and sequenced in an ABI PRISM 3100 capillary sequencer (Thermo Fisher Scientific) using the BigDye Terminator method. Sequences were then analyzed by the GENETYX version 11.0 (GENETYX, Tokyo, Japan) software.

### Phylogenetic analysis of amh sequences

The predicted aa sequences of *H. tsurugae* Amha (exons I to VII) and Amhy (exons I, IV, VI, and VII) were compared with Amh sequences of other species available in GenBank using the software GENETYX version 11.0. Multiple alignments were performed using Clustal W in MEGA software version 5.2 (Tamura *et al.* 2011). The sequences for *O. bonariensis* Amha and Amhy, *O. hatcheri* Amha and Amhy, *Dicentrarchus labrax* Amh, *Oreochromis niloticus* Amh, and *Danio rerio* Amh were used in the comparison, and *Xenopus laevis* Amh was used as the outgroup. Phylogenetic trees were generated by Neighbor-Joining (Saitou and Nei 1987), Maximum Parsimony, and Maximum Likelihood methods with 10,000 bootstrap replicates each to determine the confidence of tree topology. Analyses were performed by the MEGA software (v. 5.2).

### Sex-association analysis by amhy amplification in wild specimens

All wild-caught fish were screened for the presence of *amhy* by PCR analysis using the same primers (Table S1 in File S1; Amh 613 F and Amh 35 R) and conditions described previously. Animals carrying the *amhy* gene (*amhy* positives) were represented by *amhy*^+^ and those without it by *amhy*^−^.

### Testing of Mendelian inheritance and determination of parental genotype

To test the Mendelian inheritance of *amhy* and determine the exact parental genotype, we performed artificial insemination with gametes from four *amhy*^−^ females and four *amhy*^+^ males in single-pair crosses. Fertilized eggs from each of the crosses were incubated separately until analysis. Randomly-chosen eyed-egg stage embryos (*n* = 38–45) from each cross were analyzed by *amhy* amplification following procedures described above.

### Rearing of larvae for gene expression analysis and gonadal histology

Fertilized eggs were obtained by natural spawning using wild-caught adult individuals (48 females and 17 males) that were reared overnight in the laboratory. Fertilized eggs were collected the next morning from the bottom of the tank and incubated in flowing sea water until hatching. We could not ascertain how many females and males actually contributed fertilized eggs. Approximately 500 hatchlings (10–13 d postfertilization) were stocked in two 30 L tanks kept at 22°, the average temperature during the spawning season of *H. tsurugae* in Tateyama Bay, and reared for up to 12 wk. The tanks were supplied with filtered natural sea water at a rate of 100 ml/min. Larvae were fed rotifers *Branchionus rotundiformis* and *Artemia* *nauplii* from the first day to satiation twice daily and gradually weaned onto powdered marine fish food (AQUEON, Franklin, WI) from the fifth week of the experiment.

Fish were sampled biweekly from 0 to 10 wk after hatching (wah) for gene expression analyses and gonadal histology. The remaining larvae were sampled at the end of the rearing experiment to determine the sex ratio. The trunks of the fish were stored in RNA later (Thermo Fisher Scientific) (*n* = 8) or in Bouin’s solution (*n* = 8) for gene expression analyses and gonadal histology, respectively, at each time point. Samples in RNA later were stored at −80° until use. Bouin-fixed samples were rinsed three times with phosphate-buffered saline, transferred into 70% ethanol, and stored at 4° until use. All larvae were fin-clipped for *amhy* genotyping as described above.

### Histological analysis of gonadal sex differentiation and sex ratio

Trunk samples were dehydrated through an ascending ethanol series (70, 90, 99, and 100%), cleared in xylene, embedded in Paraplast Plus (McCormick Scientific, St. Louis, MO), sectioned serially with a thickness of 5 µm, and stained with hematoxylin and eosin. Stages of gonadal sex differentiation were determined by light microscopy using histological criteria for another atheriniform, the pejerrey *O. bonariensis* (Ito *et al.* 2005; Strüssmann and Ito 2005).

### Expression analyses by qRT-PCR and ISH

Total RNA extraction and cDNA synthesis were performed following previous studies (Yamamoto *et al.* 2014). The expression level of mRNA transcripts was analyzed by qRT-PCR using specific primers designed for *amha* and *amhy* loci. The β*-actin* gene was taken as an endogenous control because of its stability during the sex determination/differentiation period (Figure S1 and File S2). All primer sets and their respective conditions are listed in Table S1 and Table S2 in File S1.

The ISH analysis used trunks of *amhy*^+^ larvae collected before (4 wah) and after (8 wah) the onset of histological differentiation of the gonads. Ovaries from adult *amhy*^−^ specimens were used to confirm the binding specificity of the *amha*-specific probe (see the *Results* section). Samples were fixed and processed as per the protocol mentioned above. We were not able to develop an *amhy*-specific probe so hybridizations were conducted using a 775 bp *amhy* probe [nucleotides (nt) +207 to +982; exons VI to VII; and 93.5% identity with the respective sequence for *amha*] that recognized both *loci* and a 523 bp *amha*-specific probe designed in the *amha*-specific region (nt −22 to +501; exons I to III; and 17.2% of identity with *amhy*). ISH was performed as described previously (Yamamoto *et al.* 2011). Briefly, sense and antisense RNA probes were transcribed *in vitro* using digoxigenin-labeled UTP (Roche Diagnostics, Basel, Switzerland) and T7 RNA polymerase (Roche Diagnostics). Sections for ISH were permeabilized with 5 mg/ml proteinase K at 37° for 10 min. The sections were subsequently acetylated and incubated with a 0.0125–0.2 mg/ml RNA probe. After hybridization at 65° for 16 hr, the sections were washed and unbound probes were digested using 20 mg/ml RNase A to reduce background signals. The sections were then placed in blocking solution (Roche Diagnostics) at room temperature for 1 hr and incubated with the Fab fragment of an anti-DIG-alkaline phosphatase-conjugated antibody (Roche Diagnostics) diluted 1:2000 with blocking solution at 25° for 1 hr. The sections were washed and specific signals were detected by NBT/BCIP (Roche Diagnostics) according to the recommendations of the manufacturer. The signal detections by NBT/BCIP were stopped when the specific signals of antisense probes appeared.

### Statistical analysis

The significance of the association between genotype (*amhy*^+^ or *amhy*^−^) and sex phenotype, and of deviations from a 1:1 genotypic sex ratio, were determined by the Yates’ continuity corrected Chi-square test, whereas that of the differences in gene expression between groups was analyzed by ANOVA followed by the Tukey test using GraphPad Prism (v.6.0; GraphPad Software, San Diego, CA). Differences in gene expression were considered as statistically significant at *P* < 0.05.

### Data availability

DNA sequences: GenBank accessions; *H. tsurugae* Amha (KU664386) and Amhy (KU664387), *O. bonariensis* Amha (AHG98063.1) and Amhy (AAV31752.2), *O. hatcheri* Amha (AEE60845.1) and Amhy (ABF47515.2), *D. labrax* Amh (CAJ78431.1), *O. niloticus* Amh (ABS58513.1), *D. rerio* Amh (NP001007780.1), and *X. laevis* Amh (BAO04196.1).

## Results

### Isolation of amh paralogues in H. tsurugae

**Figure 1:**
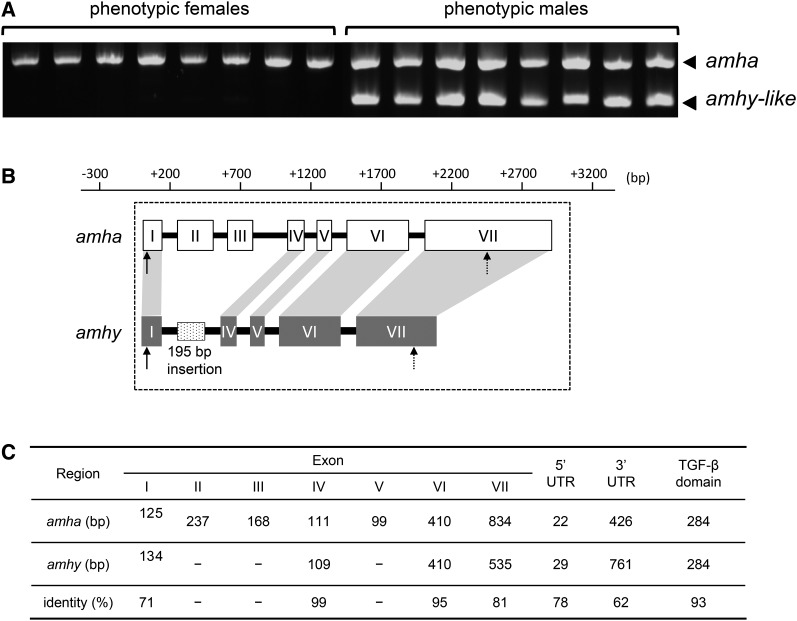
Isolation, cloning and characterization of *amha* and *amhy* in *H. tsurugae*. (A) Polymerase chain reaction-amplified *amha* in both male and female wild specimens (upper band) and *amhy* amplified only in males (lower band). (B) Comparison of full-length gene structure of *amha* and *amhy* in *H. tsurugae*. Compared to *amha*, the *amhy* gene of *H. tsurugae* is shorter, lacks exons II and III, and contains a specific insertion of 195 bp at the position of exons II and IV. Exon V is present in the genomic sequence but it is not transcribed. (C) Identity values of nucleotide sequence between *amha* and *amhy* exons, untranslated regions (UTRs), and the transforming growth factor-β (TGF-β) domain.

**Figure 2:**
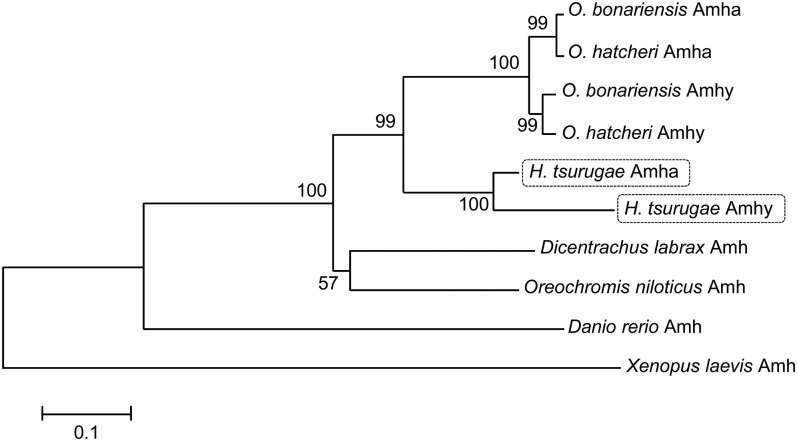
Phylogenetic analysis (neighbor-joining tree) of the amino acid sequences of *H*. *tsurugae* Amha and Amhy in relation to other species. Numbers indicate bootstrap values based on 10,000 replicates.

Two *amh* genes were cloned and isolated in *H. tsurugae*. One was detected in all individuals regardless of sex (Figure 1A) and for this reason was named *Hts-amha*, for *H. tsurugae amh* on autosomes. The cDNA sequence has 2015 nt and seven exons (Figure 1B). The other was detected only in phenotypic males (Figure 1A) and was named *Hts-amhy* for its high association with the Y chromosome as in *O. hatcheri* (Hattori *et al.* 2012) and *O. bonariensis* (Yamamoto *et al.* 2014). The full-length *Hts-amhy* cDNA sequence comprises 1838 nt and only four exons (Figure 1B). The homologs of *amha* exons II and III were absent in *amhy*. In contrast, an insertion of 195 bp was detected between exons I and IV when compared to the *amha* gene structure. The homolog of *amha* exon V was detected in genomic DNA sequence but not in cDNA sequence. The lowest and highest nt identity values were found for exons I and IV, respectively (Figure 1C). The deduced aa sequences of Amha (511 aa) and Amhy (340 aa) shared 91% of identity. Both the *amha* and *amhy* genes contained the TGF-β domain with seven canonical cysteine residues, which form disulfide bonds necessary for dimer formation. Phylogenetic analyses of Amha and Amhy aa sequences of *H. tsurugae* and other species available in the NCBI database using *X. laevis* as an outgroup revealed that *H. tsurugae amhy* and *amha* form a different clade from that of *Odontesthes* species *amhy* and *amha* (Figure 2, Figure S2 and File S2).

### Association of amhy genotype and phenotypic sex in wild and laboratory-reared fish

Adult specimens of *H. tsurugae* collected from Tokyo Bay showed high concordance between phenotypic sex and the presence/absence of *amhy*. For instance, 96% of the fish bearing testes (males) and 91.1% of the fish bearing ovaries (females) were *amhy^+^* and *amhy*^−^, respectively (Table 1; wild-caught fish; combined association of 92.6%; *P* < 0.0001). Laboratory-reared fish kept at 22° during the period of gonadal sex differentiation also showed a high association between phenotypic and genotypic sex (Table 1; laboratory-reared fish; *P* < 0.0001). In the progeny test of four single-pair crosses, the ratios of *amhy*^−^ and *amhy*^+^ in the progeny did not deviate significantly from 1:1 in any of the crosses (Table 2), supporting the Mendelian inheritance of the *amhy* gene and indicating that all males used for single-pair crosses were heterozygous (*amhy*^+/−^) for the *amhy* gene.

**Table 1:** Relationship between genotype (presence/absence of *amhy*) and the phenotypic sex in wild-caught and laboratory-reared (rearing at 22° during the period of sex determination/differentiation) *H. tsurugae*

Phenotypic Sex	Genotype		Total
	*amhy*^+^ (%)	*amhy^−^* (%)	
Wild-caught (*P* < 0.0001)*^a^*			
Testis	24 (96.0)	1 (4.0)	25 (31.9%)
Ovary	5 (8.9)	51 (91.1)	56 (69.1%)
Total	29 (35.8)	52 (64.2)	81
Laboratory-reared (*P* < 0.0001)*^a^*			
Testis	26 (96.0)	1 (4.0)	27 (57.4%)
Ovary	4 (20.0)	16 (80.0)	20 (42.6%)
Total	30 (63.8)	17 (36.2)	47

a Significant association between genotype and sex phenotype (Yates’ continuity corrected Chi-square test).

**Table 2:** Frequency of *amhy*^+^ and *amhy^−^* genotypes in progenies from four single-pair crosses of *amhy^−^* females and *amhy*^+^ males

Cross*^a^*	Genotype		Total
	*amhy*^+^ (%)	*amhy^−^* (%)	
A	21 (46.7)	24 (53.3)	45
B	16 (35.6)	29 (64.4)	45
C	21 (51.2)	20 (49.8)	41
D	20 (52.6)	18 (47.4)	38
Total	78 (46.2)	91 (53.8)	169

a The sex ratios of all progenies do not deviate significantly from 1:1.

### Expression analyses of amhy and amha during gonadal sex differentiation

**Figure 3:**
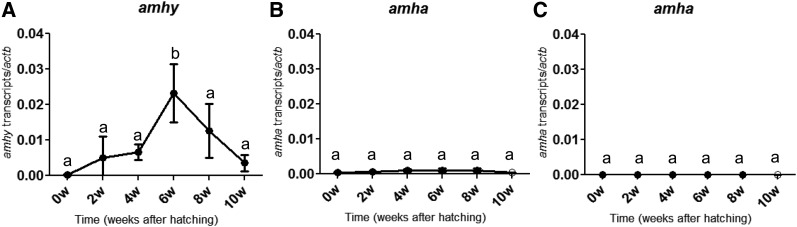
Expression profiles of *amhy* (A) and *amha* (B) in *amhy*^+^ genotype and *amha* (C) in *amhy*^−^ genotype during gonadal sex differentiation. Values represent the mean ± SEM of 3–6 fish per time point (w, weeks). Symbols with the same letter indicate groups that are not significantly different between time points.

**Figure 4:**
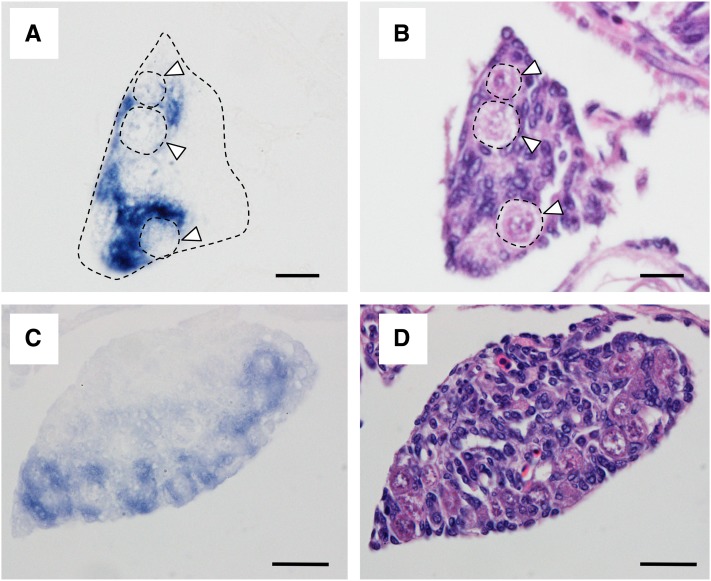
Localization of mRNAs using an *amhy* riboprobe by *in situ* hybridization in undifferentiated [4 wah (weeks after hatching), (A)] and differentiated [8 wah, (C)] gonads. Adjacent sections were stained with hematoxylin and eosin [4 wah, (B) and 8 wah, (D)]. Arrowheads indicate germ cells. Bars represent 10 μm (A and B) and 20 μm (C and D).

The qRT-PCR analysis of *amhy^+^* individuals revealed the presence of *amhy* transcripts between 2 and 10 wah with a significant peak at 6 wah (Figure 3A). In contrast, the levels of *amha* expression were extremely low in both genotypes (Figure 3, B and C). ISH signals with the *amhy* probe (that potentially also detects *amha*) were detected in undifferentiated gonads (4 wah; Figure 4A, Figure S3A and File S2) and differentiating testes (8 wah; Figure 4C) of *amhy*^+^ larvae. Signals were found in presumptive Sertoli cells surrounding germ cells at the ventral side of the gonads (Figure 4C). Since ISH signals with the *amha*-specific probe were almost undetectable in undifferentiated gonads (Figure S3B), it can be surmised by exclusion that the signals obtained at this stage with the *amhy* probe represented mostly *amhy* transcripts. This hypothesis is also supported by the qRT-PCR results described above. In addition, the binding specificity of the *amha* probe was confirmed in ovaries from adult *amhy*^−^ specimens (Figure S3D).

## Discussion

In this study, we investigated the occurrence of two *amh* paralogs and their possible roles in sex determination of the atheriniform *H. tsurugae*. One *locus* was termed *amha* for its occurrence in individuals of both sexes, whereas the other was found predominantly in males (see *Discussion* below) and for this reason was denominated as *amhy*. Although the aa sequences of both *loci* shared 91% identity, a comparative structural analysis revealed the absence of exons II, III, and V in the cDNA sequence of *amhy*, resulting in a truncated gene. Interestingly, exon V is found in the genomic DNA sequence but it is not transcribed together with other exons. The structure of the C-terminus including the TGF-β domain with seven cysteine knots, which form the disulfide bonds required for protein homodimerization (Vitt *et al.* 2001), was conserved in *amhy* *locus* and shared 93% aa identity with the same domain of *amha*. On the other hand, the N-terminus is probably not structurally complete because only exons I and IV are present. In general, biological activity in *amh* is mainly related to the C-terminus (Massagué 1990; Pfennig *et al.* 2015), but there are also cases such as human AMH in which the N-terminus actually enhances the activity of the C-terminus (Wilson *et al.* 1993). In the case of *H. tsurugae*, the integrity of the TGF-β domain suggests that *amhy* might be able to bind to the *AmhrII* (*Amh* receptor type II) and thus activate the downstream pathway of testis differentiation.

Sex-association analysis using wild adults and captive-reared juveniles (22°) showed a high but not complete association between the presence and absence of *amhy* with maleness and femaleness, respectively. The reason for the phenotype–genotype mismatches is still not clear. It could be that *amhy* is only distantly linked to the sex-determining locus and that these fish are simply recombinants. However, a more plausible explanation is that sex determination in *H. tsurugae* is affected by water temperature as in many other atheriniforms (Strüssmann and Patiño 1999; Corona-Herrera *et al.* 2016). In fact, ongoing experiments have provided preliminary evidence of thermal modulation of sex determination in this species (K. Miyoshi, C.A. Strüssmann, and Y., Yamamoto, unpublished data), which in other atheriniforms has been shown to cause phenotypic–genotypic mismatches (Yamamoto *et al.* 2014).

The analysis of mRNA expression during larval development showed that *amhy* transcripts were restricted to *amhy^+^* individuals. The expression of *amhy* was detected from before the appearance of the first signs of histological sex differentiation in presumptive Sertoli cells surrounding germ cells in the undifferentiated gonad and was maintained during testis differentiation. In contrast, *amha* showed low, basal expression levels in both genotypes during the same period. This is similar to the pattern described for *O. hatcheri* (Hattori *et al.* 2012) and different to that of *O. bonariensis*, where *amha* is coexpressed with *amhy* during the critical period of sex determination (Yamamoto *et al.* 2014). It has been reported that sex determination in *O. bonariensis* shows higher temperature sensitivity than that of *O. hatcheri* (Strüssmann *et al.* 1997). The high thermosensitivity at both high and low temperatures in the former species could be related to the profile of *amha*, which increases before the appearance of sex-specific histological differences not only in XY genotypes but also during masculinization of XX individuals (Yamamoto *et al.* 2014). If this is true, one might expect only moderate effects of temperature on sex ratios in *H. tsurugae* as is the case of *O. hatcheri*. Studies on the effects of temperature on the sex ratios and expression profiles of *amha* and *amhy* of *H. tsurugae* are currently underway.

Although these results suggest that *amhy* is a candidate sex determination gene in *H. tsurugae* as in other atheriniforms, it is not yet a foregone conclusion that this gene is an ortholog of the *amhys* of *O. hatcheri* and *O. bonariensis*. On the contrary, so far the results of phylogenetic analysis place it in a separate clade with other *amhys*, suggesting that it may have evolved independently. Sex-determining genes are known to show recurrent and independent evolution in teleosts (*e.g.*, as exemplified in medaka species; Matsuda *et al.* 2002; Myosho *et al.* 2012; Takehana *et al.* 2014). In fact, recent reports implicating *amh/AMH* as a candidate sex-determining gene in unrelated taxa such as tilapia *O. niloticus* (Eshel *et al.* 2014; Li *et al.* 2015) and the marsupial mammal *Ornithorhynchus anatinus* (Cortez *et al.* 2014) lend support to the notion that this gene has a high probability of being recruited as a key genetic player of sex determination. Hence, it is possible that a coincidental *de novo* appearance of *amhy* has occurred in *H. tsurugae*. Nevertheless, the hypothesis of conservation cannot be fully ruled out at this point. For example, given the particularly fast evolutionary rates of sex-determining genes in relation to their autosomal paralogues (Mawaribuchi *et al.* 2012) and the relatively large genetic distance of Atherinopsidae and Atherinidae families (Bloom *et al.* 2012), it could be that the *amhys* in *H. tsurugae* and *Odontesthes* have accumulated enough structural changes as to make them lose the characteristics they once had in common. The absence of some exons in the truncated *amhy* of *H. tsurugae* could be such a case. This issue will only be clarified by analyzing relevant sequences for other atheriniform families besides Atherinopsidae and Atherinidae.

In conclusion, this study demonstrated that *amhy*, although with some structural differences in relation to the *amhy* of some New World atheriniforms (Hattori *et al.* 2012; Yamamoto *et al.* 2014), is also present in an Old World atheriniform. The high expression of *amhy* early in larval development and the high association with maleness in captive-reared and wild animals make *H. tsurugae amhy* a sex-determining gene candidate. The finding of a putative Y chromosome-specific marker will be extremely useful for monitoring the effects of environmental factors and anthropogenic influences on sex determination in this species. For example, ongoing studies suggest the occurrence of TSD in *H. tsurugae* (K. Miyoshi, C.A. Strüssmann, and Y. Yamamoto, unpublished data), which might place this species at higher risk of sex reversal and skewed sex ratios within a scenario of global warming and/or climate change. Moreover, species presenting both temperature-dependent and genotypic sex determination mechanisms such as *H. tsurugae* may be suitable as early-warning bioindicators of the effects of global warming on fish reproduction.

## Supplementary Material

Supplemental material is available online at www.g3journal.org/lookup/suppl/doi:10.1534/g3.117.042697/-/DC1.
