# Supplementary figures and images for: A Duplicated, Truncated amh Gene Is Involved in Male Sex Determination in an Old World Silverside
Source: G3 (Bethesda). 2017 Jun 13;7(8):2489–95. doi: 10.1534/g3.117.042697 (PMC5555456; doi:10.1534/g3.117.042697)

## Slide 1
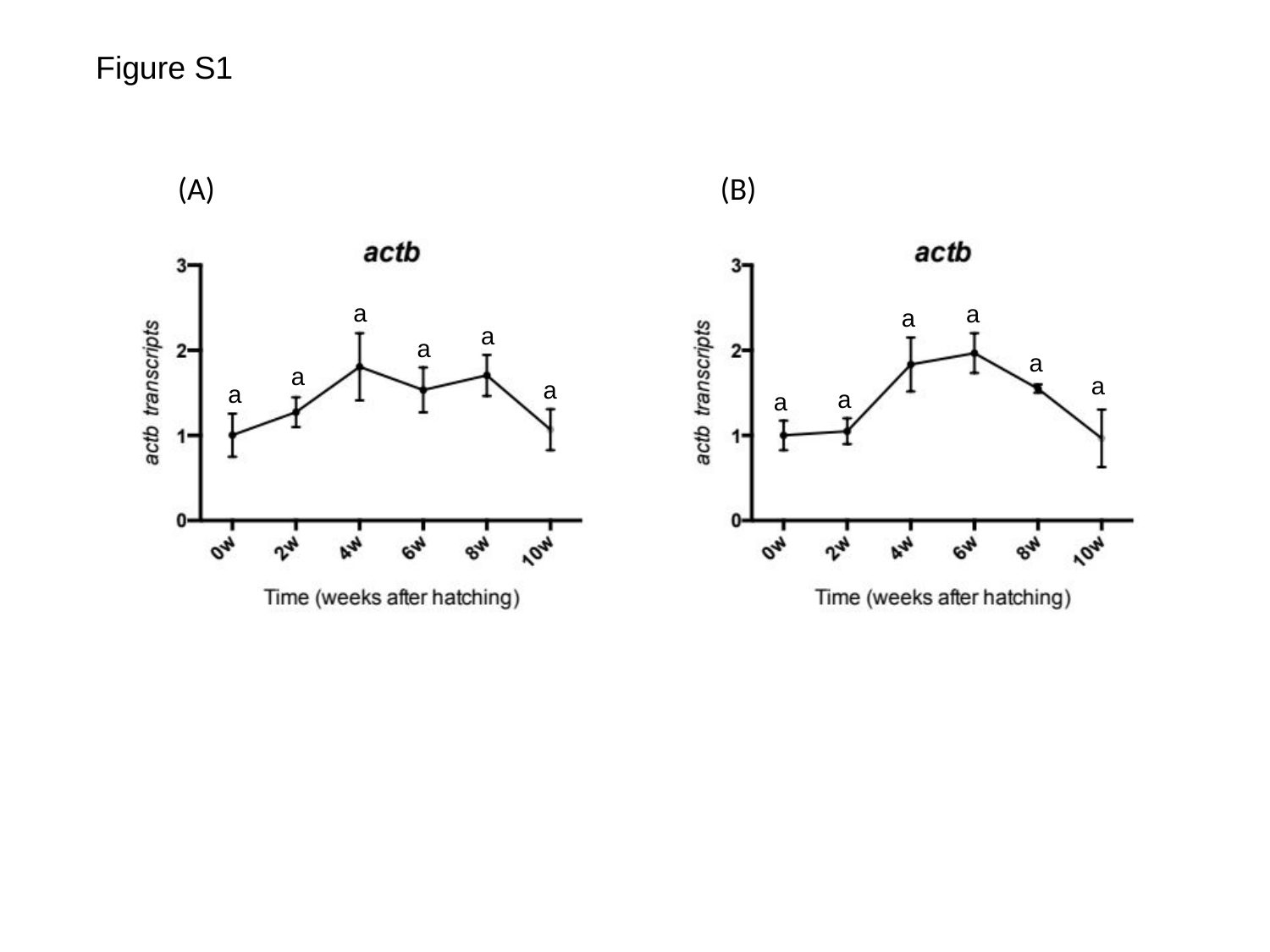

Figure S1
(A)
(B)
a
a
a
a
a
a
a
a
a
a
a
a

Supplement: Supplementary file 1 [file 2489FigureS1.pptx]

## Slide 1
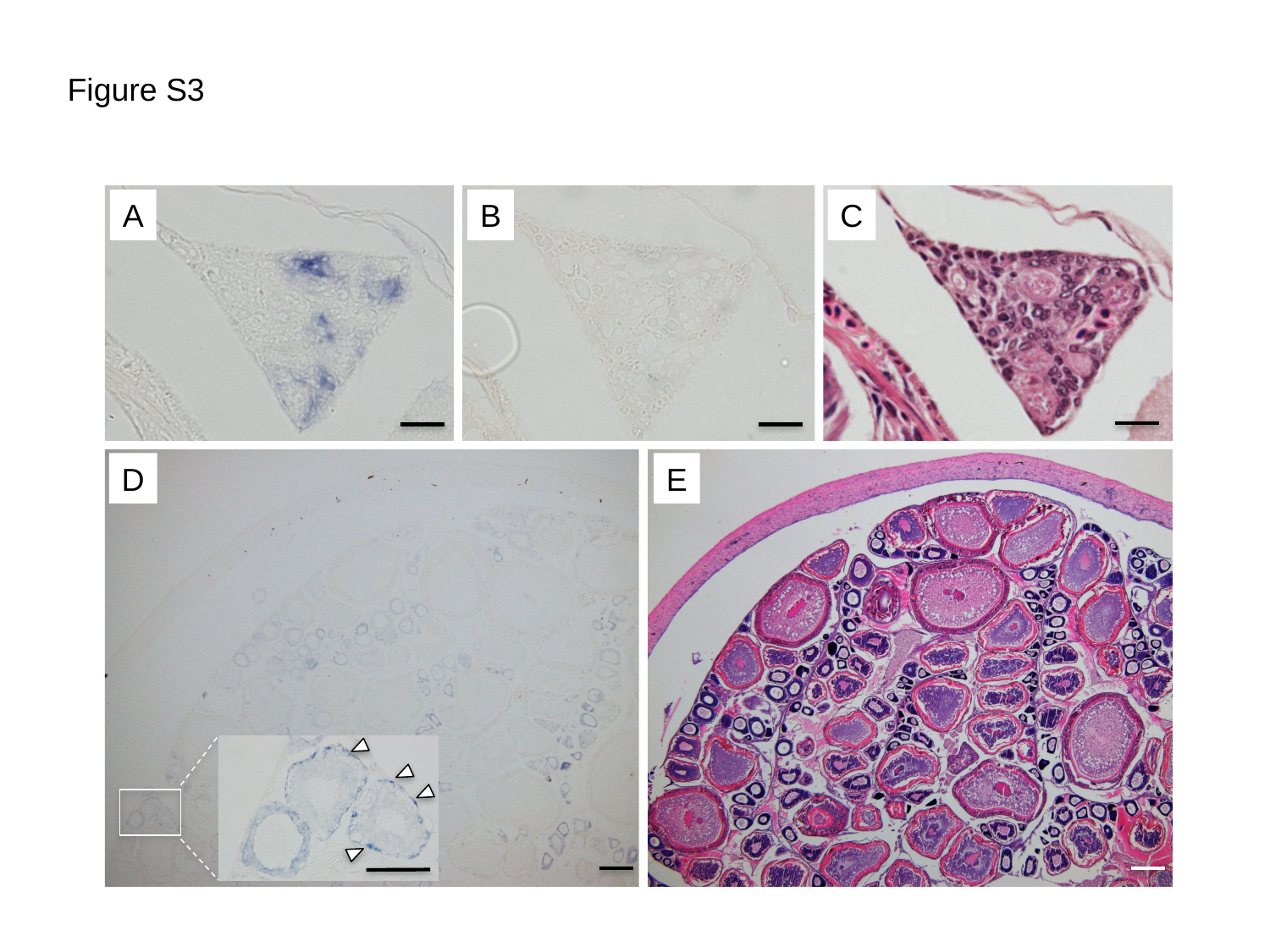

Figure S3
A
B
C
D
E

Supplement: Supplementary file 3 [file 2489FigureS3.pptx]
