# Supplementary material for: A Duplicated, Truncated amh Gene Is Involved in Male Sex Determination in an Old World Silverside
Source: G3 (Bethesda). 2017 Jun 13;7(8):2489–95. doi: 10.1534/g3.117.042697 (PMC5555456; doi:10.1534/g3.117.042697)
Supplement: Supplementary file 2 [file 2489FigureS2.pptx]

## Slide 1
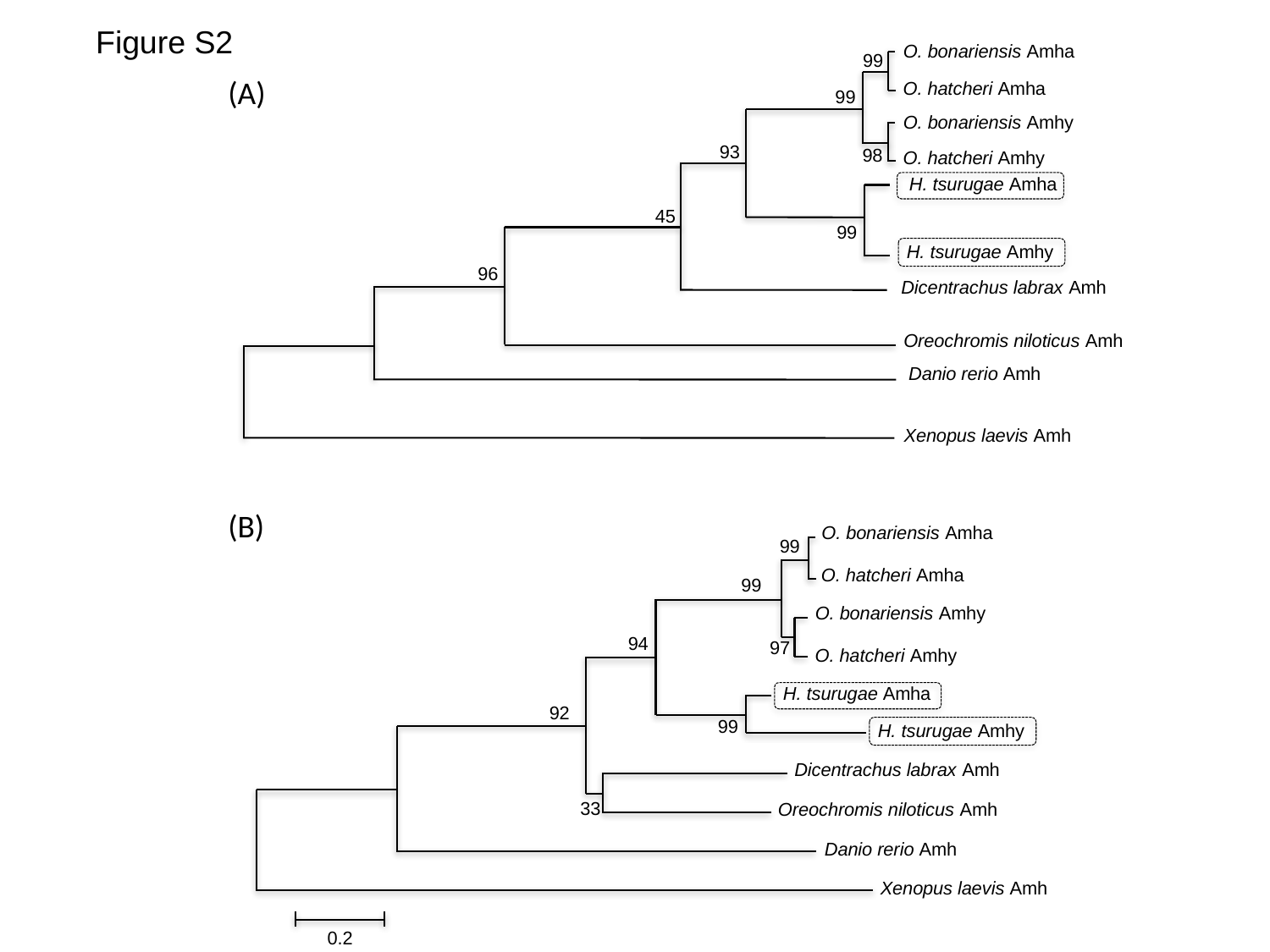

Figure S2
O. bonariensis Amha
99
99
93
98
45
99
96
(A)
O. hatcheri Amha
O. bonariensis Amhy
O. hatcheri Amhy
H. tsurugae Amha
H. tsurugae Amhy
Dicentrachus labrax Amh
Oreochromis niloticus Amh
Danio rerio Amh
Xenopus laevis Amh
(B)
O. bonariensis Amha
99
O. hatcheri Amha
99
O. bonariensis Amhy
94
97
O. hatcheri Amhy
H. tsurugae Amha
92
99
H. tsurugae Amhy
Dicentrachus labrax Amh
33
Oreochromis niloticus Amh
Danio rerio Amh
Xenopus laevis Amh
0.2
