# Supplementary material for: A Duplicated, Truncated amh Gene Is Involved in Male Sex Determination in an Old World Silverside
Source: G3 (Bethesda). 2017 Jun 13;7(8):2489–95. doi: 10.1534/g3.117.042697 (PMC5555456; doi:10.1534/g3.117.042697)
Supplement: Supplementary file 4 [file 2489FileS1.doc]

**Table S1.** Sequence of primers used for isolation and analysis of *amha* and *amhy* genes in *Hypoatherina tsurugae*.

| Purpose | Primers | Sequence | Primer order |
| --- | --- | --- | --- |
| *amha* Degenerate PCR | Amh 208 F | 5’-ACGGTGCTCTCCTTCACTT-3’ | Sense |
|  | Amh 2R | 5’-GTCTKCAGVGCCTTCAGCAG-3’ | Antisense |
| *amha* Genome walking / 5’ RACE | 5end Amha Race R1 (1st PCR) | 5’-GACATCCACACTCCCTTGCTA-3’ | Antisense |
|  | 5end Amha Race R2 (nested) | 5’-CCACCTCTTCCTCATTTATCAACTCC-3’ | Antisense |
| *amha* 3’ RACE | 3end Amha Race F1 | 5’-AGACACATCAAGGGTT-3’ | Sense |
|  | 3 end Amha Race F2 | 5’-CCCCACTATCTTCTCCTTCAC-3’ | Sense |
| *amhy* Genotyping | Amh 613 F | 5’-CTCACAGCCCTGCAGTGT-3’ | Sense |
|  | Amh 35 R | 5’-AGAAGGTCTTTCAGGTTTTGCT-3’ | Antisense |
| *amhy* Genome walking | GW Amhy F1 | 5’-CGAGGACGCAGGTTACATTGG-3’ | Sense |
|  | GW Amhy F2 | 5’-TTTGCAACATCTATGGAATATATTGTTG-3’ | Sense |
|  | GW Amhy R1 | 5’-ACAACTTCACAAATCACTCTAAGAAATG-3’ | Antisense |
|  | GW Amhy R2 | 5’-ACTTTCACCATAAACAGATTTCTTTGGA-3’ | Antisense |
| *amhy* 5’ RACE | 5 end Amhy Race R1 | 5’-CACCGTCTGCAGGGCCTTCAGCA-3’ | Antisense |
|  | 5 end Amhy Race R2 | 5’-AGCCTCTCTACGGCTTTCTG-3’ | Antisense |
| *amhy* 3’ RACE | 3 end Amhy Race F1 | 5’-GAGCACGGCATGGATTTCGG-3’ | Sense |
|  | 3 end Amhy Race F2 | 5’-AGTCTCAGCTGATACAGGTGGACT-3’ | Sense |
| *amha* qRT-PCR | AmhaRT355F | 5’-AACAGCAGTACTGGTGTCAG-3’ | Sense |
|  | AmhaRT607R | 5’-CCATGTCTGCTCCACGTTTCC-3’ | Antisense |
| *amhy* qRT-PCR | AmhyRT236F | 5’-CCAGTTTGGACACATCAAGGGTT-3’ | Sense |
|  | AmhyRT394R | 5’-CTGGAGGATAAACCGAGAGTCAA-3’ | Antisense |
| *actb* qRT-PCR | Beta actin RT F | 5’-GTGCTGTCTTCCCCTCCATC-3’ | Sense |
|  | Beta actin RT R | 5’-TCTTGCTCTGGGCTTCATCA-3’ | Antisense |
| *amh* ISH Probe | ISH_amh_Fw | 5’- CCAGTTTGGACACATCAAGGGTT-3’ | Sense |
|  | ISH_amh_Rv | 5’- TGGAGAGAAAGGCGCCTTGT-3’ | Antisense |
| *amha* ISH Probe | Amha specific 1F | 5’- GCATTCAAGCGGACAGCAA-3’ | Sense |
|  | Amha specific 523R | 5’- GTGAGGCTGCAGACACTGAC-3’ | Antisense |

**Table S2.** PCR conditions and primer combinations used for isolation and analysis of *amha* and *amhy* genes in *Hypoatherina tsurugae*.

| Purpose | Sense primer | Antisense primer | Denaturing temperature (time) | Annealing temperature (time) | Extension temperature (time) | Cycles |
| --- | --- | --- | --- | --- | --- | --- |
| *amha* Degenerate PCR | Amh 208 F | Amh 2R | 94°C (60s) | 60°C (60s) | 72°C (180s) | 35 |
| *amha* 5’Genome walking | AP1 | 5end Amha Race R1 (1st PCR) | 94°C (60s) | 60°C (60s) | 72°C (180s) | 35 |
|  | AP2 | 5end Amha Race R2 (nested) | 94°C (60s) | 68°C(180s) | 72°C (300s) | 35 |
| *amha* 5’ RACE | UPM | 5end *amha* Race R1 (1st PCR) | 94°C (60s) | 60°C (60s) | 72°C (180s) | 35 |
|  | NUP | 5end *amha* Race R2 (nested) | 94°C (60s) | 68°C (60s) | 72°C (150s) | 35 |
| *amha* 3’ RACE | 3end *amha* Race F1 (1st PCR) | UPM | 94°C (60s) | 60°C (60s) | 72°C (180s) | 35 |
|  | 3 end *amha* Race F2 (nested) | NUP | 94°C (60s) | 68°C (60s) | 72°C (150s) | 35 |
| *amhy* Genotyping | *amh* 613 F | *amh* 35 R | 94°C (60s) | 60°C (60s) | 72°C (180s) | 35 |
| *amhy* 3’Genome walking | GW Amhy F1 (1st PCR) | AP1 | 94°C (60s) | 60°C (60s) | 72°C (180s) | 35 |
|  | GW Amhy F2 (nested) | AP2 | 94°C (60s) | 68°C (180s) | 72°C (300s) | 35 |
| *amhy* 5’Genome walking | AP1 | GW Amhy R1 (1st PCR) | 94°C (60s) | 60°C (60s) | 72°C (180s) | 35 |
|  | AP2 | GW Amhy R2 (nested) | 94°C (60s) | 68°C (180s) | 72°C (300s) | 35 |
| *amhy* 5’ RACE | UPM | 5 end Amhy Race R1 (1st PCR) | 94°C (60s) | 60°C (60s) | 72°C (180s) | 35 |
|  | NUP | 5 end Amhy Race R2 (nested) | 94°C (60s) | 68°C (60s) | 72°C (150s) | 35 |
| *amhy* 3’ RACE | 3 end Amhy Race F1 (1st PCR) | UPM | 94°C (60s) | 60°C (60s) | 72°C (180s) | 35 |
|  | 3 end Amhy Race F2 (nested) | NUP | 94°C (60s) | 68°C (60s) | 72°C (150s) | 35 |
| *amha* qRT-PCR | AmhaRT355F | AmhaRT607R | 94°C (30s) | 60°C (30s) | 68°C (90s) | 30 |
| *amhy* qRT-PCR | AmhyRT236F | AmhyRT394R | 94°C (30s) | 60°C (30s) | 68°C (90s) | 30 |
| *actb* qRT-PCR | Beta actin RT F | Beta actin RT R | 94°C (30s) | 60°C (30s) | 68°C (90s) | 30 |
| *amh* ISH Probe | ISH_amh_Fw | ISH_amh_Rv | 94°C (30s) | 60°C (30s) | 68°C (90s) | 35 |
| *amha* ISH Probe | Amha sp.1F | Amha sp.523R | 94°C (30s) | 60°C (30s) | 68°C (90s) | 35 |

AP1, AP2, UPM, and NUP are universal primers supplied in the respective kits.
