# Supplementary material for: A Duplicated, Truncated amh Gene Is Involved in Male Sex Determination in an Old World Silverside
Source: G3 (Bethesda). 2017 Jun 13;7(8):2489–95. doi: 10.1534/g3.117.042697 (PMC5555456; doi:10.1534/g3.117.042697)
Supplement: Supplementary file 5 [file 2489FileS2.docx]

**Figure Legends (Supplemental Materials)**

Figure S1. Expression profiles of *actb* in *amhy*+ (A) and *amhy*− (B) genotypes during gonadal sex differentiation. Values represent the mean ± SEM of 3-6 fish per time point. Symbols with the same letter indicate groups that are not significantly different between time points.

Figure S2. Alternative phylogenetic trees generated with the Maximum Parsimony (A) and Maximum Likelihood (B) methods based on the amino acid sequences of *H*. *tsurugae* Amha and Amhy and Amh of other species. Numbers indicate bootstrap values based on 10,000 replicates.

Figure S3. Localization of mRNAs using an *amhy* (A) and *amha* (B and D) riboprobe by ISH in undifferentiated gonad (4 wah) and adult ovary. Adjacent sections were stained with hematoxylin and eosin (C and E). Arrowheads indicate *amha* signals in the follicle cell layer. Scale bars represent 10 μm (A, B, C), 200 μm (D, E) and 100 μm (inset in D).
